# Hypothesized mechanisms of death in swimming: a systematic review

**DOI:** 10.1186/s13102-023-00799-w

**Published:** 2024-01-02

**Authors:** Yunheng Yao, Michael A. DiNenna, Lili Chen, Shirong Jin, Sixian He, Jinshen He

**Affiliations:** 1 Department of Orthopaedic Surgery, Third Xiangya Hospital of Central South University, Changsha, 410013 Hunan China; 2 Xiangya School of Medicine, Central South University, Changsha, 410013 Hunan China; 3 Department of Mechanical and Material Science Engineering, University of Pittsburgh, Pittsburgh, PA 15213 USA

**Keywords:** Swimming, Death, Mechanism, Cardiovascular disease, Drowning

## Abstract

**Background:**

The study aims to update the specific classification of mechanisms of death in swimming and to demonstrate these categories are reasonable, by analyzing more characteristics of death cases, evaluating the available evidence and determining their quality.

**Methods:**

Original articles were queried from PubMed, Web of Science, Embase databases, Cochrane Library, and Scopus. Included studies, which were evaluated as level 4 evidence or higher according to the Oxford Centre for Evidence-Based Medicine, discussed hypothesized mechanisms of death in swimming. Parameters analyzed in this study included decedents’ characteristics, outcome measures, findings, methodological index for non-randomized studies (MINORS), and critical evaluation of each study classified by death mechanism.

**Results:**

A total of twenty-five studies were included for further analysis: fourteen were associated with cardiovascular diseases, two were about cerebrovascular diseases, two contained respiratory diseases, seven were about hazardous conditions and three contained other drownings, which provided evidence for mechanisms of death.

**Conclusions:**

It is found that cardiovascular disease is the main cause or contributing factor of death in swimming. Respiratory diseases and cerebrovascular diseases are difficult to be definitive mechanism categories due to insufficient evidence. Hazardous conditions appear to be one of the possible risk factors because there are more cases of deaths from unsafe environments in swimming, but further statistics and research are still needed to support this view. Our study may have important implications for developing potential prevention strategies for sports and exercise medicine.

**Trial registration:**

PROSPERO ID (CRD42021267330). Registered Aug 13th 2021.

**Supplementary Information:**

The online version contains supplementary material available at 10.1186/s13102-023-00799-w.

## Background

Swimming and triathlons have become increasingly popular activities. Between 2009 and 2015, annual participation in triathlons rose from 120,620 to 196,303 (63%) in the United Kingdom [1]. A 2020 study investigated children's swimming ability, in which descriptive statistics showed that over 50% of children were able to perform some basic swimming skills [2]. According to existing studies, swimming is an exercise modality that is highly suitable for health promotion and disease prevention, such as cognitive functioning, the treatment of major depressive disorder, cerebral palsy and other aspects [3–6]. But swimming has also been found to cause injuries and even death. The majority of the deaths (95%) in triathlon competitions occur during the swim phase [7].

To prevent death in swimming, there have been some efforts to summarize the cause of death in swimming. The possible causes of death in swimming have been summarized in a study, including exertional heat stroke, heart attack, long QT syndrome, pulmonary edema, knocked unconscious, and panic attack [8]. The study did not generalize and classify different mechanisms from the causes of death, and the sample size for each cause of death was insignificant. And one study published in 2016 summarized possible mechanisms as hypothermia/hyperthermia, cardiac arrhythmias/abnormalities and pulmonary edema, on the basis of which, they analyzed the outcome measures, findings, and critical evaluation for each case [9]. But this systematic review concluded that limited evidence does not support these as common death mechanisms. A new study classified the pre-existing medical conditions of fatal or non-fatal drowning as diseases of the nervous system, mental and behavioral conditions and diseases of the circulatory system [10].

The study aims to update the possible classification of mechanisms of death in swimming, by evaluating the available evidence, whose quality is determined utilizing the Grading of Recommendations Assessment, Development and Evaluation, and analyzing the data of gender, age, location of the decedents to demonstrate that these categories are reasonable. These works provide a better understanding of swimming-related deaths, which may have implications in developing potential prevention strategies. We hypothesized that death mechanisms in swimming are cardiovascular diseases, Cerebrovascular diseases, respiratory diseases and hazardous conditions, among which, cardiovascular diseases are the main mechanism.

## Methods

### Study selection

Articles were comprehensively retrieved via online database searching, a review of reference lists and cited reference searches. The online databases used were PubMed, Web of Science, Embase databases, Scopus, and Cochrane Library. The retrieved articles were limited to human-subject literature published from 1990 to Jan 1st, 2023. The following search terms were used: (“swim*” OR “swimming*” OR “triathlon*”) AND (“death*” OR “mortality*”) AND (“drowning*” OR “pulmonary edema” OR “hyperthermia*” OR “hypothermia*” OR “sudden death” OR “cardiac arrhythmia*” OR “sudden cardiac death”).

### Inclusion and exclusion criteria

This study included articles that met the following inclusion criteria: (1) studies discussed hypothesized mechanism of death in swimming; and (2) level 4 evidence or higher (case report, case series, case control studies, retrospective analysis or randomized clinical trial) according to the Oxford Centre for Evidence-Based Medicine.

Irrelevant articles and studies that failed to meet inclusion criteria, including (1) articles studied deaths in diving and cold-water immersion (if not specifically related to swimming); and (2) studies investigating outcomes of death in baths, were excluded.

### Data extraction and analysis

Two authors (YY, LC) reviewed papers identified in searches, assessing potentially eligible studies for inclusion and exclusion criteria. Type of article, grade of evidence reported outcomes, and available data were extracted independently. Any disagreements were resolved with discussion and additional review before final analysis by the professor (JH). No attempts were made to contact any of the authors to request additional data. Statistical analysis was feasible after summarizing comparable outcomes between studies.

Parameters analyzed in this study included 1) outcome measures, findings, and critical evaluation of each study classified by death mechanism; 2) gender, age, location of where the decedent died in swimming. And the methodological index for non-randomized studies (MINORS) checklist was used to assess the methodologic quality of the included non-randomized controlled studies (Supplemental Table 1).

### Patient and public involvement

No patient involved. Neither patients nor the public were involved in the design, conduct, reporting, or dissemination of the research, as it was not feasible or appropriate for this systematic review. Our review had registered on PROSPERO and was assigned with registration number: CRD42021267330, which is publicly available at https://www.crd.york.ac.uk/prospero/display_record.php?RecordID=267330.

## Results

**Fig. 1:**
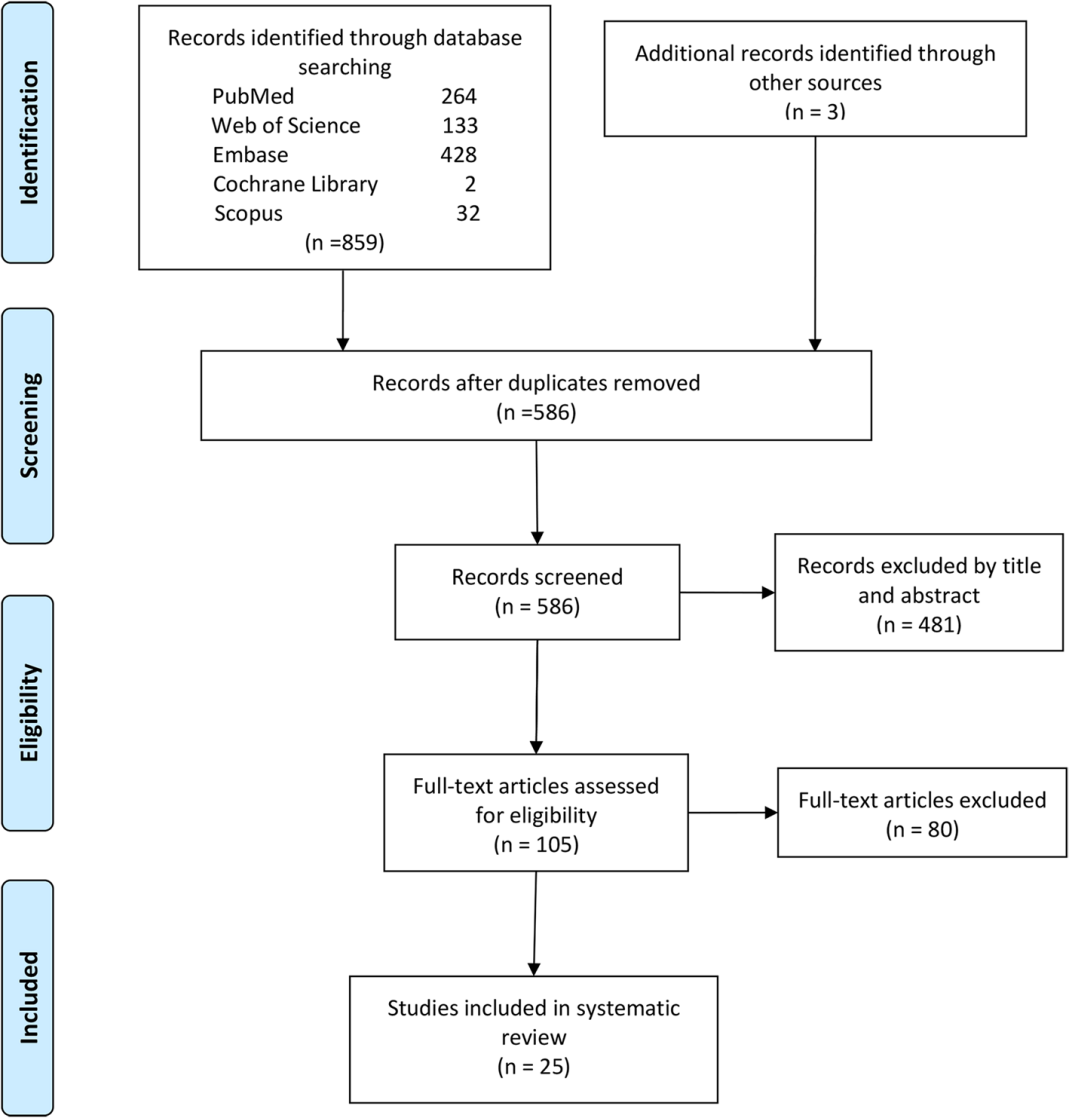
PRISMA (Preferred Reporting Items for Systematic Meta-Analyses) flow diagram

The flow of the literature search is presented in Fig. 1. The initial search identified 859 articles, including records identified through database searching and other sources. A total of 586 articles remained after duplicates were excluded. After the examination of titles and abstracts for relevance, 105 articles remained and full texts were assessed for eligibility. A total of 25 articles met all eligibility criteria investigating different death mechanisms during swimming (Table 1).

**Table 1:** Summary of the characteristics of the different death mechanisms in swimming

**Characteristic**	**Cardiovascular diseases (*n* = 89)**	**Cerebrovascular diseases** **(*n* = 2)**	**Respiratory diseases (*n* = 5)**	**Hazardous conditions (*n* = 13)**	**Others (*n* = 91)**
Sex	(*n* = 62)	(*n* = 2)	(*n* = 4)	(*n* = 9)	(*n* = 91)
Male	49	2	4	6	61
Female	13	0	0	3	30
Age					
Mean ± SD	35.8 ± 25.4	54.5 ± 9.2	18.0 ± 0	29.1 ± 21.7	7.4 ± 9.6
Range	11–82	48–61	17–22	12–66	1–57
Body of water	(*n* = 29)	(*n* = 1)	(*n* = 1)	(*n* = 7)	(*n* = 73)
pool	4 (13.8%)	0 (0%)	0 (0%)	3 (42.9%)	58 (79.5%)
Lake/reservoir	3 (10.3%)	0 (0%)	1 (100%)	2 (28.6%)	15 (20.5%)
Ocean/harbor	18 (62.1%)	1 (100%)	0 (0%)	2 (28.6%)	0 (0%)
River	4 (13.8%)	0 (0%)	0 (0%)	0 (0%)	0 (0%)

### Cardiovascular diseases

Fourteen studies involving potential cardiac causes of swimming-related death met the criteria for inclusion (Table 2) (Supplemental Table 2). Among them, four studies of death in swimming have concluded that cardiovascular disease is the main cause or contributing factor of death [1, 11–13]. The most common cardiovascular abnormalities is atherosclerotic coronary heart disease, in addition, arterial hypertension, chronic myopericarditis, cardiac aneurysm of the left ventricle and so on [14–16]. Most cases had varying degrees of coronary atherosclerosis, myocardial hypertrophy and myocardial scar, sometimes secondary to pulmonary edema (Table 3).

**Table 2:** Studies on cases of death from cardiovascular diseases in swimming

**Author**	**Year**	**Type of study**	**LOE**	**Findings**	**Critical evaluation/comments**
Windsor	2020	Case Series (*n* = 2)	4	Cardiovascular pathology was found to be a cause or contributing factor in half of all deaths	The irregularity of recording procedures and the limitation of time led to a smaller sample size and a higher death rate
Harris	2017	Case Series (*n* = 27)	4	27 of 61 decedents with autopsy reports (44%) had clinically relevant cardiovascular abnormalities, most frequently atherosclerotic coronary disease(*n* = 18)	Incomplete identification and underestimated events. Pre-race medical history is unknown in most cases
Kurosu	2016	Case Series (*n* = 1)	4	Anomalous origin of coronary artery (AOCA) was a rare cause of death in sporting	The ages investigated were limited to 31 years and below
Škavić	2015	Case Series (*n* = 1)	4	The death rates in elderly Croatian women due to swimming reached 0.25	The number of cases was too small and limited information was available at autopsy
Anästhesie	2015	Case Report (*n* = 1)	4	Abnormal left coronary artery (ALCA) causes sudden cardiac death	The cause of coronary artery abnormalities is unknown
Durakovic	2012	Case Series (*n* = 17)	4	The leading cause of death from swimming is organic heart disease	It is not easy to define exactly what is an exertion-related death
Duraković	2011	Case Series (*n* = 6)	4	CHD in elders is a usual underlying cause of sudden cardiac death during immediately after physical activity	More efficient medical screening systems will be needed
Cedrone	2010	Case Report (*n* = 1)	4	Hypertrophic obstructive cardiomyopathy (HOCM) is an risk factor for sudden cardiac death (SCD) in young adults	The incidence of SCD in young people is not high enough for extensive, wide-scale examinations
Tester	2009	Case Series (*n* = 28)	4	Nearly 30% of the victims of swimming-related drowning hosted a cardiac channel mutation	The difference in mutation detection rates among drowning victims of different ages was not statistically significant, possibly due to the small sample size
Durakovic	2008	Case Report (*n* = 1)	4	The cause of death for that man was myocarditis	In specific cases, radionuclide studies, cardiac catheterization and magnetic resonance imaging may be needed
Duraković	2004	Case Report (*n* = 1)	4	The mechanism of these five exercise-induced sudden death events may be malignant ventricular arrhythmia	It is difficult to define exactly what sudden death caused by exercise is
Ackerman	1999	Case Report (*n* = 1)	4	The postmortem identification of a novel mutation in the ion-channel gene KVLQT1, causing the long-QT syndrome, in a sample of the woman’s myocardium	It is not yet known whether mutations in cardiac ion channels underlie a substantial number of unexplained drownings
Myrianthefs	1997	Case Report (*n* = 1)	4	Ventricular arrhythmias and sudden cardiac death caused by exercise appears to be inherited in an autosomal dominant manner and can be triggered by swimming	With only one family case, the sample is too small to draw conclusions
Janataa	1994	Case Report (*n* = 1)	4	Congenital anomaly (an anomalous origin of the left coronary artery) should be considered in cases of major cardiac events in young people	The exact etiology and mechanism of sudden death are not known

According to the Oxford Centre for Evidence-Based Medicine 's classification of "evidence", Case-series or Case–control studies, or Poor Quality Prognostic Cohort studies should be classified as Level 4 evidence

*LOE* level of evidence

**Table 3:** Data on deaths from cardiovascular diseases during swimming

**No**	**Age (years)**	**Sex**	**Event**	**Findings at autopsy**	**LVH (mm)**	**Heart enlargement**	**Reference**
1	11	male	lake	AOCA	-	-	Anästhesie 2015
2	12	female	-	Ventricular arrhythmia and polymorphic ventticular tachycardia	-	-	Myrianthefs 1997
3	16	female	-	AOCA	-	-	Janataa 1994
4	17	male	-	AOCA	-	-	Kurosu 2016
5	18	male	sea	Chronic pericarditis, left ventricular fibrosis, anterior wall tumor of the LV	-	-	Durakovic 2012
6	18	male	-	Chronic pericarditis, myocardial scar, aneurysm of the LV, coronaries with no changes	-	-	Duraković 2004
7	18	male	-	Chronic pericarditis, left ventricular aneurysm	-	-	Durakovic 2008
8	19	female	pool	Myocardial ischemia, QT prolongation	-	-	Ackerman 1999
9	20	male	river	HOCM	-	-	Cedrone 2010
10	29	male	pool	Coronaries with no changes	25	-	Durakovic 2012
11	33	male	sea	Left ventricular hypertrophy	15	-	Durakovic 2012
12	42	male	-	Coronary atherosclerosis, 70% to 75% stenosis of the proximal LADCA, borderline cardiac hypertrophy	-	yes	Windsor 2022
13	45	male	lake	Myocardial ischemia, myocardial hypertrophy, hypertensive heart disease	-	-	Windsor 2020
14	47	male	sea	Moderate to severe coronary atherosclerosis	-	-	Durakovic 2012
15	50	male	river	Coronary atherosclerosis with coronary artery stenosis	22	yes (500 g)	Durakovic 2012
16	52	male	river	Coronary atherosclerosis, all coronary arteries stenosis up to 70%	18	yes (350 g)	Durakovic 2012
17	57	male	lake	Coronary atherosclerosis, diffuse myocardial fibrosis, myocardial adipose tissue infiltration	-	-	Durakovic 2012
18	57	male	sea	Severe coronary atherosclerosis, AMI in the posterior wall of the LV	15	-	Durakovic 2012
19	63	male	sea	Extensive atherosclerosis, diffuse myocardial fibrosis	25	yes (580 g)	Durakovic 2012
20	68	male	pool	Diffuse coronary atherosclerosis, > 75% stenosis of the LADCA	19	yes	Durakovic 2012
21	68	-	pool	CHD, critical stenosis LADCA, large scar of the LV	20	yes	Duraković 2011
22	69	male	sea	Moderate diffuse coronary artery atherosclerosis, myocardial scarring on the anterior wall	-	-	Durakovic 2012
23	69	male	sea	Moderate systemic atherosclerosis	yes	yes (500 g)	Durakovic 2012
24	69		sea	CHD, myocardial scar anterior wall	18	yes	Duraković 2011
25	74	male	sea	Severe coronary atherosclerosis, myocardial scarring of the posterior wall	19	-	Durakovic 2012
26	74	male	sea	Coronary atherosclerotic stenosis < 1 mm, myocardial scarring on the posterior wall	21	-	Durakovic 2012
27	74	-	sea	CHD generalized, myocardial scars posterior wall	-	-	Duraković 2011
28	82	male	sea	Moderate diffuse coronary atherosclerosis, diffuse myocardial fibrosis	19	-	Durakovic 2012
29	82	male	sea	Diffuse myocardial fibrosis, moderate to severe coronary atherosclerosis, myocardial scarring of the anterior wall on the LV	-	-	Durakovic 2012
30	82	female		Coronary atherosclerosis, myocardial scarring of the posterior wall	22	-	Durakovic 2012
31	82	-	sea	CHD, myocardial scars, chronic pericarditis	23	yes	Duraković 2011
32	82	-	sea	Myocardial fibrosis, pericardial adhesions, coronaries with no changes	19	yes	Duraković 2011
33	82	-	sea	CHD, myocardial scar anterior wall, diffuse myocardial fibrosis	-	-	Duraković 2011
34	82	female	sea	Coronary artery stenosis, generalized atherosclerosis predominating in the coronary artery, myocardium scarring in the posterior lateral wall of the left lung	22	-	Škavić 2015

*LVH* left ventricle hypertrophy, *AOCA* Abnormal Coronary Artery Origin, *LV* left ventricle, *HOCM* Hypertrophic obstructive cardiomyopathy, *AMI* acute myocardial infarction, *LADCA* left descending anterior coronary artery, *CHD* coronary heart disease

Some studies suggested that congenital or inherited cardiovascular abnormalities are contributing risk factors for hazardous events in swimming in many cases, especially for young people. In a study of 28 people who drowned while swimming, it was found that pathogenic mutations in critical ion channel genes may be present in nearly 30% of swim-related drowning cases, which were associated with potentially fatal arrhythmia syndromes known as long QT syndrome (LQTS) and catecholaminergic polymorphic ventricular tachycardia (CPVT) [17]. And a study of a 19-year-old female swimmer, who died of hypoxic encephalopathy after near-drowning, found that her myocardium samples showed a novel mutation in the ion channel gene KVLQT1, which causes long QT syndrome, and thus drowned in swimming [18]. In another study of a family, in which 3 members had ventricular arrhythmias and sudden cardiac death caused by exercise, including a 12-year-old girl who died during a swimming competition, it was found that this disease appears to be inherited in an autosomal dominant manner and can be triggered by swimming [19]. And other four studies, based on deaths in young people while swimming, concluded that congenital anomalies, such as anomalous origin of coronary artery (AOCA), should be considered in cases of major cardiac events in young people [20–22], and hypertrophic obstructive cardiomyopathy (HOCM) was a risk factor for sudden cardiac death in young adults [23].

### Cerebrovascular diseases

Two articles meeting the inclusion criteria respectively mentioned one death from cerebrovascular disease in middle-aged men, one of whom reported drowning due to cerebral, cerebellar, and subarachnoid hemorrhage, accompanied by cardiac enlargement, left ventricular hypertrophy and diffuse coronary atherosclerosis, while the other was confirmed diffuse cerebral infarction, cerebral hypoxia and arrhythmia while swimming (Table 4) (Supplemental Table 3) [1, 12].

**Table 4:** Studies on cases of death from cerebrovascular diseases in swimming

**Author**	**Year**	**Type of study**	**LOE**	**Findings**	**Critical evaluation/comments**
Windsor	2020	Case Series (*n* = 1)	4	A 48-year-old male was found dead while swimming from diffuse cerebral infarction, cerebral anoxia and cardiac arrhythmia	The irregularity of recording procedures and the limitation of time led to a smaller sample size and a higher death rate
Durakovic	2012	Case Series (*n* = 1)	4	An elderly man has drowned after suffering a stroke while swimming. The autopsy results involved bleeding of the brain, cerebellum and subarachnoid space, and lesions of the cardiovascular system	It is not easy to define exactly what is an exertion-related death

### Respiratory diseases

Two studies involving swimming related deaths due to respiratory diseases met the inclusion criteria (Table 5) (Supplemental Table 4). A study of 16 drowning cases, 4 of which died of dangerous underwater breath-holding behaviors (DUBBs), including intentional hyperventilation, static apnea, and hypoxic training [24]. Another study showed that a healthy 18-year-old male swimmer who suffered cardiopulmonary arrest while swimming in a freshwater lake is suspected to have died of secondary acute respiratory distress syndrome (ARDS) [25].

**Table 5:** Studies on cases of death from respiratory diseases in swimming

**Author**	**Year**	**Type of study**	**LOE**	**Findings**	**Critical evaluation/comments**
Boyd	2015	Case Series (*n* = 4)	4	All of the 4 cases were caused by DUBBs, including intentional hyperventilation, static apnea, and hypoxic training	There may be some missed cases because of limited resources of surveillance data
Diamond	2011	Case Report (*n* = 1)	4	Acute respiratory distress syndrome (ARDS) has the potential for early onset and rapid progression in the setting of submersion	Evidence-based bypass algorithms for some acute medical conditions, such as resuscitated submersion victims, is not established

### Hazardous conditions

Eleven articles met the inclusion criteria, which studied cases of death from dangerous circumstances while swimming (Table 6) (Supplemental Table 5). In a study, a boy died from electrocution while swimming and diving [26]. A boy in another study died from drowning after being paralyzed by an electrical current from a defective pool light [27]. In two articles, there were respectively a 12-year-old girl [28] and a 13-year-old boy [29] who were trapped in a large, uncovered suction drainage hole and death had occurred due to drowning, which may be also related to traumatic shock caused by the suction mechanism. Decedents in two articles died from several distinct patterns of injury caused by crocodile attacks in swimming, including completely traumatic ruptures of the body, avulsions of limbs, punctures, ecchymosis, pulmonary congestion, edema, and so on [30, 31]. In a study of cold water swimming, two deaths in an official ice swimming competition was mentioned, whose cause of death from cold water swimming was an initial neurogenic cold shock response, or the initial neurogenic cold shock response or a progressive decrease in swimming efficiency or hypothermia [32]. In addition, drunkenness may lead to death in swimming, supported by two included cases [12, 14].

**Table 6:** Studies on cases of death from hazardous conditions in swimming

**Author**	**Year**	**Type of study**	**LOE**	**Findings**	**Critical evaluation/comments**
Knechtle	2020	Case Series (*n* = 2)	4	The likely cause of death from cold water swimming is an initial neurogenic cold shock response, or hypothermia	Cold water swimming is practiced by a very small number of extreme athletes, and poses significant health risks to inexperienced and untrained swimmers
Atilgan	2020	Case Report (*n* = 1)	4	The girl drowned after her feet were vacuumed into a drainage hole in a swimming pool	The cause of death was drowning due to a faulty drainage system in the swimming pool
Focardi	2019	Case Report (*n* = 1)	4	The boy died due to drowning, who was trapped at the abdomen by an uncovered suction drainage hole	
Sinton	2016	Case Series (*n* = 5)	4	At autopsy, several distinct patterns of injury were observed, including a complete traumatic rupture of the body	It is difficult to determine from an autopsy whether the crocodile attack occurred before or after death
Škavić	2015	Case Series (*n* = 1)	4	A 66-year-old woman was found dead of drunkenness (acute alcohol poisoning) while swimming, accompanied by systemic atherosclerosis, left ventricular hypertrophy, liver diseases and trauma	The number of cases was too small and limited information was available at autopsy
Durakovic	2012	Case Series (*n* = 1)	4	The teenager died while swimming in a state of level 2 to 3 intoxication. Autopsy revealed a 450 g enlarged heart, pulmonary edema, and bilateral pleural effusion	It is not easy to define exactly what is an exertion-related death
Harding	2006	Case Report (*n* = 1)	4	Crocodile attacks result in numerous skin lacerations, punctures and ecchymosis, accompanied by pulmonary congestion and edema, resulting in death	Although the death was attributed to drowning based on pulmonary edema, multiple sharp force injuries were listed as contributing factors
CDC	1996	Case Series (*n* = 1)	4	Contact with electricity can result in death through temporary paralysis and drowning of persons who are swimming or diving	No state or national surveillance systems exist for related deaths. And electricity-related drownings are difficult to identify
Goodson	1993	Case Report (*n* = 1)	4	The boy died from drowning after being paralyzed by electrical current from a defective pool-light	No evidence of electrical trauma was found on the body

### Others

Three studies involving drowning deaths in swimming due to other or unknown causes met the inclusion criteria (Table 7) (Supplemental Table 6). The absence of formal swimming courses and delayed intervention are also contributing conditions to increase swimming mortality. A case–control study of 88 children and adolescents aged 1 to 19 years who died of unintentional drowning found a protective association between past participation in formal swimming lessons and the risk of drowning in children [33]. A 50-year-old female swimmer died of postanoxic brain damage, multiorgan failure syndrome, and other reasons following drowning due to delayed intervention by rescue teams after drowning [34]. Another study reported two drowning deaths from unknown causes during a 750-m swim [1].

**Table 7:** Studies on cases of death from drowning due to other or unknown causes in swimming

**Author**	**Year**	**Type of study**	**LOE**	**Findings**	**Critical evaluation/comments**
Windsor	2020	Case Series (*n* = 2)	4	Two unexplained drowning deaths were reported during the 750 m swim	The irregularity of recording procedures and the limitation of time led to a smaller sample size and a higher death rate
Brenner	2009	Case–Control Study (*n* = 88)	3	Participation in formal swimming lessons was associated with an 88% reduction in the risk of drowning in the 1- to 4-year-old children	Small sample size, especially in the older age group
Broi	2009	Case Report (*n* = 1)	4	The cause of death was assessed as postanoxic brain damage, multiorgan failure syndrome and cardiac failure following drowning	Improper rescue surgery was considered to be more related to the woman's injury than to her death

## Discussion

Our study identified cardiovascular disease as the most important mechanism of death in swimming [35]. Cardiovascular disease was mentioned in nearly half (12 / 23) of the studies that met the inclusion criteria, and four studies concluded that cardiovascular disease was responsible for the majority of deaths in swimming. The most common cause of accidental death in young competitive athletes is a cardiac illness, usually that of congenital etiology. Many seemingly healthy people who suffer fatal drowning while swimming may be due to severe cardiac arrhythmias, which are caused by congenital long-QT syndrome (LQTS) and catecholaminergic polymorphic ventricular tachycardia (CPVT). In patients with LQTS, swimming has been identified as a relative gene-specific arrhythmia trigger for LQTS1 type (LQTS1) caused by KCNQ1 mutations, the exact mechanism of which is unclear. The possible explanation is that the diving response during swimming triggers parasympathetic driven bradycardia, while cold shock activates sympathetic division of the autonomic nervous system, resulting in tachycardia, which constitutes "autonomic conflict". LQT1 patients with IKs channel dysfunction due to KCNQ1 mutations are less efficient than normal in shortening the QT interval during tachycardia. This lack of proper QT adaptation leads to possible apical torsion ventricular tachycardia [36]. Catecholaminergic Polymorphic Ventricular Tachycardia is an inherited arrhythmogenic disorder of myocardial calcium hemostasis, autosomal dominant form and autosomal recessive form of which may be due to mutations in the ryanodine receptor and calmodulin 2 genes, respectively. Certain adrenergic triggers, such as swimming, predispose CPVT patients to sudden death via bidirectional polymorphic ventricular tachycardia [37]. Among the variable types of abnormal coronary artery origin, the AOCA passing between the pulmonary artery and the aorta, either the left coronary artery arising from the right coronary sinus (ALCA) or the right coronary artery arising from the left coronary artery (ARCA), carries a high risk of sudden death due to exercise load. The possible mechanism is that the coronary artery becomes squeezed by passing between an expanded PA and the aorta, so that the blood flow through the coronary artery becomes reduced during intense exercise, which leads to the occurrence of myocardial ischemia [21]. ARCA is four times more common than ALCA, but ALCA is a more common cause of sudden cardiac death than ARCA, because left ventricular ischemia from left main occlusion is usually more life-threatening than right coronary occlusion [22]. And based on a hemodynamic analysis of the anomalous origin of the right coronary artery (RCA) from the left coronary artery sinus (AORL), the entrance cross-sectional area of AORL is reduced, leading to a decrease in the volume flow and pressure of RCA, which can be used as a numerical guide for clinical diagnosis of AORL ischemia symptoms [38]. Among aerobic exercises, swimming is associated with more catecholamine-induced tachycardia and increased stroke volume and tachycardia, which is also a risk factor for fatal functional outflow tract obstruction in patients with resting hypertrophic cardiomyopathy, leading to fatal ventricular arrhythmias [23]. Overall, swimming deaths associated with cardiovascular disease vary with the age of the population, such as in young people the main risk factors are the AOCA, HOCM, and so on, while CHD is more considered in sports in the elderly [13, 21, 23]. The associated mortality highlights the importance of early athlete screening and identification of existing heart disease.

Respiratory diseases are thought to be not completely reliable to the hypothesized swimming death mechanisms because few cases are supporting this mechanism. The included respiratory failure death cases are related to two concepts, DUBBs and ARDS [24, 25]. Hyperventilation or breath-holding before diving or swimming reduces the body's storage of CO_2_ and carbon dioxide partial pressure (PCO_2_) to delay the brain's response to surface breathing which is an effective technique to trick their bodies into delaying the stimulation of breathing, thereby improving swimmers' performance. However, the suppression of respiration also reduces the partial pressure of oxygen (PO2) in arterial blood, resulting in fatal hypoxia and loss of consciousness underwater, namely breath-hold blackout caused by DUBBs [24]. In different sports disciplines, swimming seems to have a higher incidence of Exercise-Associated Hyponatremia (EAH) than cycling, mountain biking, running and other events, especially in long-distance open water swimming. EAH progressively worsens during endurance exercise, and respiratory diseases, including respiratory arrest, occurs when plasma sodium concentrations are reduced to < 110–115 mmol/L, which can eventually lead to death [39]. In addition, swimming-induced pulmonary edema (SIPE) is characterized by rapid onset of shortness of breath, cough, and rales, which can be fatal if the athlete continues to compete [40]. The increase in cardiac volume during swimming, on the one hand, increases the myocardial oxygen demand, on the other hand, also elevates the left ventricular end-diastolic pressure in heart that are noncompliant from previous myocardial infarction, which in turn increases the pulmonary venous pressure and may explain the cause of dyspnea [41]. In conclusion, more relevant studies should be conducted in the future to further investigate the injury mechanisms associated with respiratory diseases in swimming.

Deaths from hazardous environmental factors in swimming, such as electrical casualties in swimming pools and malfunctioning drainage systems and so on, are important public health issues, but the physiological mechanisms involved are poorly studied. Compared to the typical "entrance-exit" wound pattern when exposed to electricity in dry conditions, swimming pool electrical damage appears to lack significant external tissue damage, but rather cardiac symptoms are more commonly seen, that is, arrhythmias of varying degrees, ranging from essentially asymptomatic to severe and even fatal [42]. But according to the above case, another possible mechanism is that the muscles exposed to the low-voltage current become paralyzed, leaving the victim unable to escape the power source and drowning [27]. Trauma caused by inhalation by swimming pool drain, including multiple organ congestion, abdominal contusion and so on, is not fatal in fact, so the direct factor of death is still drowning [28, 29]. Deaths from crocodile attacks can involve a combination of blunt or sharp force injuries, blood loss, and drowning, as the trauma can be very severe, including almost complete fragmentation, head and chest crushing injuries, and limb avulsion, resulting in blunt craniocerebral trauma, cascading chest, and rapid death from exsanguination. Severe sepsis can also occur, as a variety of microorganisms have been found in the mouth of crocodiles, such as Salmonella, Aeromonas hydrophila, Clostridium, and others. But the exact role of each factor and the causal relationship between trauma and drowning may not be clear [30, 31]. The risk of death in the first stage of cold water swimming is mainly derived from the neurogenic cold shock response, while when immersed for a short period of time, the physical challenges are mainly related to musculoskeletal damage caused by neuromuscular cooling, disrupted nerve conduction and increased nociceptive sensitivity (pain receptors), which can lead to a physiological state to peripheral paralysis and, as a result, may increase the risk of drowning. If athletes swim in cold water for too long, they may experience hemostasis due to hypothermia, lactic acidosis, vascular insufficiency, cognitive impairment, and arrhythmia [32]. Cold water endurance swimming may affect the lungs of healthy leisure triathletes for up to 2.5 h after swimming, and some people seem to be more prone to lung injury than others [43]. From the data available, the concentration-effect relationship between alcohol and drowning while boating, swimming, or engaging in any other water activity is apparent, and mechanisms may include dose-dependent psychomotor impairment, lowering of the cognitive processes, and increased risk-taking behavior [44]. Mortality and cardiac arrest rates were 3.3 times higher among male participants over 40 years of age than among men under 40 years of age, and the risk increased with each additional decade of age after 40 [11]. In conclusion, the hazardous condition is not an exact pathological mechanism, but rather a low probability risk factor, which may involve multiple possible mechanisms.

Drowning is a leading cause of unintentional injuries and deaths worldwide [45]. Univariate analysis showed that men and rural residents consistently had much higher drowning mortality rates than women and urban residents, which occurred mainly in natural water and during the summer season [46, 47]. Especially for children under 4 years of age, drowning is the most common cause of accidental injury and death [45]. Participation in formal swimming lessons was associated with an 88% reduction in the risk of drowning in the 1- to 4-year-old children, while training of parents and supervisors in post-drowning prevention and first aid may also go some way to avoiding accidents [33, 48]. Especially in water-rich countries such as the Netherlands, timely swimming safety education for the children and families of recent immigrants to help them adapt may be an important measure [49]. Survivors of drowning often experience severe neurological disorders, such as children with ABI from drowning suffer from a selective differentiation syndrome, in which motor deficits are largely responsible for their inability to convey intact cognition and perception. But overall, the consequences of drowning in neuropathological studies have not been fully established [34, 45, 50].

Drownings during swimming can also be the result of panic attacks, which are accompanied by a complete loss of swimming ability. Competitive swimmers can panic when swimming in open water because they face a different environment than an Olympic pool and need to use a different stroke than the one they trained for. Open-water swimmers can experience similar panics when suddenly confronted with icy water, rip currents, or unexpected underwater objects. The mechanism may be due to: an extreme sympathetic nervous system over-activation causes a combination of physical and psychological stress during panic in water, which may aggravate cold shock, cause paralysis or loss of muscle strength, and cause complete or severe loss of swimming ability. It can eventually lead to accidental drowning or non-drowning death [51].

The epidemiology of exercise-related sudden cardiac death has also been implicated in recent studies. In Spain, sports-related sudden cardiac death has a very low incidence, in which the most frequent sports disciplines are football (49%), gymnastics (15%), and running (12%) [52, 53]. And the main causes of sudden cardiac death are ischemic heart disease (63%), cardiomyopathies (21%), and sudden arrhythmic death syndrome (6%) [52]. A large sampling of Scottish and England population-based cohort, the association of six different types of sports/exercises with the risk of all-cause and cardiovascular disease (CVD) mortality was investigated. In studies on the long-term health effects of specific sports, CVD mortality involved in swimming (HR = 0.59, 95% CI 0.46 to 0.75), racquet sports (HR = 0.44, 95% CI 0.24 to 0.83) and aerobics (HR = 0.64, 95% CI 0.45 to 0.92) were significantly reduced, with no significant effects on cycling, running, and football. These findings suggest that participation in specific sports may have significant public health benefits [54]. And the three major causes of sudden death in organized school sports in Japan were sudden cardiac arrest, head trauma, and heat related injury, accounting for 77.2% of all reported cases [55]. Myocardial inflammation is a common cause for the onset of cardiovascular disease and sudden cardiac death in athletes. Physical exercise may be seen as an acute trigger of myocardial ischemia or arrhythmias in some susceptible individuals [56].

Taken together, in order to reduce the death caused by various factors in swimming, it is recommended to make a full range of preparations before swimming, during swimming and after drowning and strive to update relevant facilities. Before swimming, all subjects wishing to perform moderate to high intensity exercise are advised to undergo pre-participation screening and annual follow-up, including thorough preparticipation physical examination and indicated diagnostic tests, which may help in avoiding the fatal event [16, 57]. For example, echocardiography is an effective method to detect congenital cardiovascular abnormalities [16, 21]. In order to ensure the safety of the swimming environment, the swimming pool staff should eliminate the hidden dangers of the swimming pool lighting and drainage system, and swimmers in the field should ensure that there are no sharks, rapids and other dangerous factors in the environment before entering the water [26–31]. During exercise, swimmers are advised to wear a portable monitor to monitor their heart rate to prevent sudden illness such as sudden cardiac death. The development of portable monitors that can detect electrocardiograms will also be beneficial. It will be better if the monitor or sensor can be connected with automatic life-saving system and shore helper wirelessly, or call for help to prompting rescue automaticly. In addition, improvements are necessary for emergency rescue systems for drowning people in triathlon or other swimming environments, such as providing flotation devices to swimmers via drones or using new self-inflatable Quick Rescue (QR) flotation device [58, 59]. Municipalities and jurisdictions are advised to consider preventing DUBBs by enacting public health education and regulations to reduce the associated fatal and non-fatal injuries in swimming [24]. In general, in addition to the invention, maintenance and update of protective facilities, the realization of swimming safety depends more on the safety awareness and protection level of swimmers themselves, so the publicity and education of swimming safety for professional swimmers and the public is crucial. And water safety competency assessments should be universal, not only in swimming pools but also in open water, to ensure that swimmers know whether they have the ability to spot potential hazards and stay safe in different water environments [60].

Several limitations of this study should be mentioned. First, due to the limitations of research method design, the lack of international collaboration may lead to potential publication bias. In addition, since the database we selected is Pubmed and other international databases with a wide range of use, in fact, we did not search the literature in some domestic databases of non-native English speaking countries such as China (China National Knowledge Infrastructure), Russia (eLibrary), and France (Cairn), which may lead to our omission of non-English studies in literature inclusion. Second, due to the imperfect detection system and the absence of autopsy reports, some cases of deaths in swimming have not been reported or analyzed in depth, which results in too small sample sizes for some mechanisms. In some cases, the information of the age, sex, and place of death was incomplete, which leads to some omissions in the statistics. Third, the risk factors mentioned above are not necessarily the actual fatal cause, as it is difficult to determine from an autopsy whether it occurred during or after swimming. Future research needs to be done through closer international collaboration, such as recruiting players from World Aquatics or other international organizations for swimming as research subjects or collecting data from them [61]. Additional cases with full autopsy reports will be studied to determine whether respiratory diseases and hazardous environments are reliable mechanisms and to explore the potential neurohumoral, cardiovascular, metabolic or respiratory mechanisms involved. Besides, researches related to updating prevention strategies, such as drowning detection and emergency rescue systems, are also necessary.

## Conclusion

**Fig. 2:**
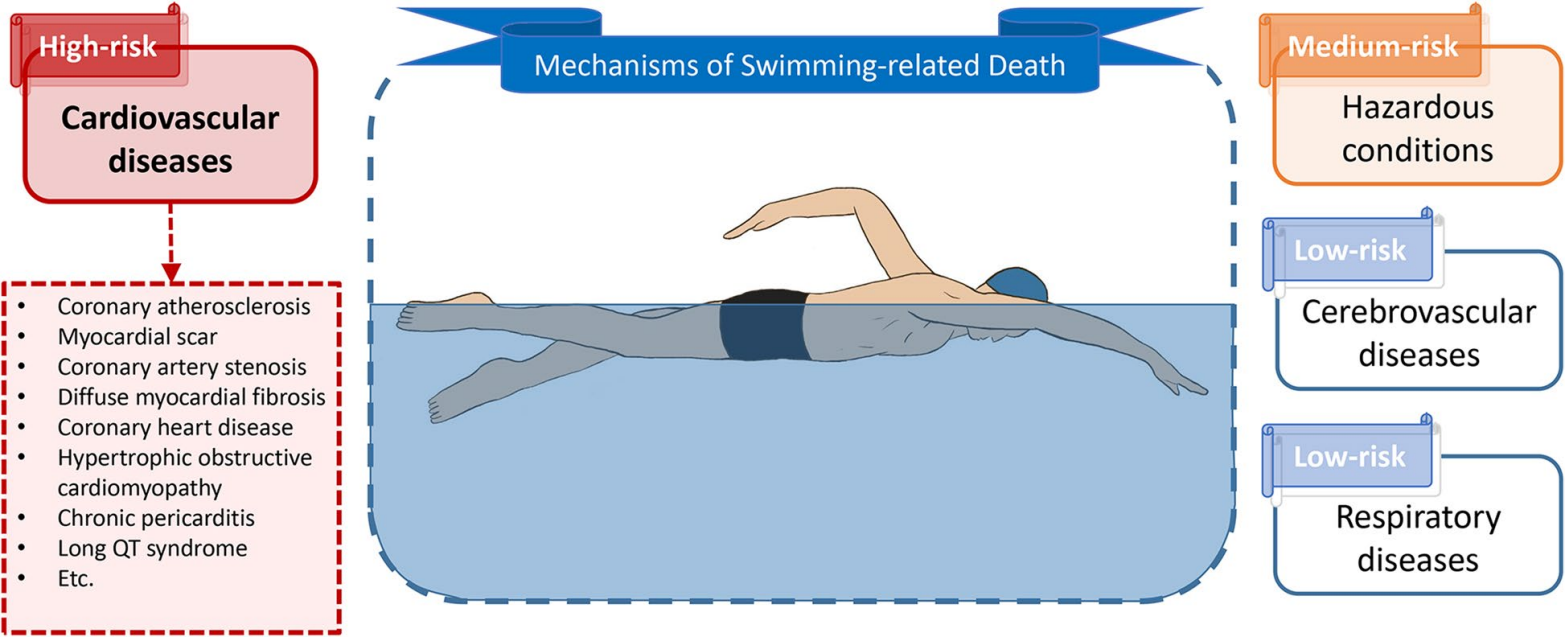
Mechanisms of swimming-related death

The included studies support possible death mechanisms (Fig. 2). Cardiovascular diseases are the main cause or contributing factor of death in swimming, while hazardous conditions appear to be medium-risk mechanisms, as well as cerebrovascular diseases and respiratory diseases are relatively low-risk. However, there are still some limitations, including the small sample size of cases, which leads to the failure of in-depth analysis of some mechanisms, and the absence of detailed autopsy reports, especially ones that contain detailed information about future age, sex and place of death. Our study summarizes several possible mechanisms of death in swimming, which may have implications in developing potential prevention strategies in sport and exercise medicine.

### Supplementary Information

**Additional file 1: Supplemental Table 1. **The MINORS Score for All Non-Randomized Controlled Studies. **Supplemental Table 2. **Characteristics of Swimmers Who Died of Cardiovascular Diseases. **Supplemental Table 3. **Characteristics of Swimmers Who Died of Cerebrovascular Diseases. **Supplemental Table 4. **Characteristics of Swimmers Who Died of Respiratory Diseases. **Supplemental Table 5. **Characteristics of Swimmers Who Died of Hazardous Conditions. **Supplemental Table 6. **Characteristics of Swimmers Who Died of Drowning Due to Other or Unknown Causes.

**Additional file 2: **PRISMA 2020 Checklist.

## Data Availability

The datasets used and/or analyzed during the current study are available from the corresponding author on reasonable request.
