# Supplementary material for: Hypothesized mechanisms of death in swimming: a systematic review
Source: BMC Sports Sci Med Rehabil. 2024 Jan 2;16:6. doi: 10.1186/s13102-023-00799-w (PMC10763430; doi:10.1186/s13102-023-00799-w)
Supplement: Supplementary file 1 — Additional file 1: Supplemental Table 1. The MINORS Score for All Non-Randomized Controlled Studies. Supplemental Table 2. Characteristics of Swimmers Who Died of Cardiovascular Diseases. Supplemental Table 3. Characteristics of Swimmers Who Died of Cerebrovascular Diseases. Supplemental Table 4. Characteristics of Swimmers Who Died of Respiratory Diseases. Supplemental Table 5. Characteristics of Swimmers Who Died of Hazardous Conditions. Supplemental Table 6. Characteristics of Swimmers Who Died of Drowning Due to Other or Unknown Causes. [file 13102_2023_799_MOESM1_ESM.docx]

**Supplemental Table 1** The MINORS Score for All Non-Randomized Controlled Studies

| **Study** | **A clearly stated aim** | **Inclusion of consecutive patients** | **Prospective collection of data** | **Endpoints appropriate to the aim of the study** | **Unbiased assessment of the study endpoint** | **Follow-up period appropriate to the aim of the study** | **Loss of follow up less than 5%** | **Prospective calculation of the study size** | **Additional criteria in comparative studies** | | | | **Total** |
| --- | --- | --- | --- | --- | --- | --- | --- | --- | --- | --- | --- | --- | --- |
|  |  |  |  |  |  |  |  |  | **An adequate control group** | **Contemporary groups** | **Baseline equivalence of groups** | **Adequate statistical analyses** |  |
| **Cardiovascular diseases** | | | | | | | | | | | | | |
| Windsor 2020 | 2 | 1 | 1 | 1 | 1 | 0 | 0 | 1 | NA | NA | NA | NA | 7 |
| Harris 2017 | 2 | 2 | 2 | 2 | 1 | 0 | 0 | 2 | NA | NA | NA | NA | 11 |
| Kurosu 2016 | 2 | 0 | 0 | 2 | 1 | 0 | 0 | 0 | NA | NA | NA | NA | 5 |
| Škavić 2015 | 2 | 1 | 1 | 1 | 1 | 0 | 0 | 1 | NA | NA | NA | NA | 7 |
| Anästhesie 2015 | 2 | 0 | 0 | 1 | 1 | 0 | 0 | 0 | NA | NA | NA | NA | 4 |
| Durakovic 2012 | 2 | 1 | 1 | 1 | 1 | 0 | 0 | 1 | NA | NA | NA | NA | 7 |
| Duraković 2011 | 2 | 1 | 1 | 2 | 1 | 0 | 0 | 1 | NA | NA | NA | NA | 8 |
| Cedrone 2010 | 2 | 0 | 0 | 1 | 1 | 0 | 0 | 0 | NA | NA | NA | NA | 4 |
| Tester 2009 | 2 | 1 | 1 | 2 | 1 | 0 | 0 | 1 | NA | NA | NA | NA | 8 |
| Durakovic 2008 | 2 | 0 | 1 | 1 | 1 | 0 | 0 | 1 | NA | NA | NA | NA | 6 |
| Duraković 2004 | 2 | 0 | 1 | 1 | 1 | 0 | 0 | 1 | NA | NA | NA | NA | 6 |
| Ackerman 1999 | 2 | 0 | 2 | 2 | 1 | 0 | 0 | 2 | NA | NA | NA | NA | 9 |
| Myrianthefs 1997 | 2 | 0 | 2 | 2 | 1 | 0 | 0 | 2 | NA | NA | NA | NA | 9 |
| Janataa 1994 | 2 | 0 | 0 | 1 | 1 | 0 | 0 | 0 | NA | NA | NA | NA | 4 |
| **Cerebrovascular diseases** | | | | | | | | | | | | | |
| Windsor 2020 | 2 | 1 | 1 | 1 | 1 | 0 | 0 | 1 | NA | NA | NA | NA | 7 |
| Durakovic 2012 | 2 | 1 | 1 | 1 | 1 | 0 | 0 | 1 | NA | NA | NA | NA | 7 |
| **Respiratory diseases** | | | | | | | | | | | | | |
| Boyd 2015 | 2 | 1 | 1 | 2 | 1 | 0 | 0 | 1 | NA | NA | NA | NA | 8 |
| Diamond 2011 | 2 | 0 | 0 | 2 | 1 | 0 | 0 | 0 | NA | NA | NA | NA | 5 |
| **Hazardous conditions** | | | | | | | | | | | | | |
| Knechtle 2020 | 2 | 2 | 1 | 2 | 1 | 0 | 0 | 1 | NA | NA | NA | NA | 9 |
| Atilgan 2020 | 2 | 0 | 0 | 1 | 1 | 0 | 0 | 0 | NA | NA | NA | NA | 4 |
| Focardi 2019 | 2 | 0 | 0 | 1 | 1 | 0 | 0 | 0 | NA | NA | NA | NA | 4 |
| Sinton 2016 | 2 | 1 | 1 | 1 | 1 | 0 | 0 | 1 | NA | NA | NA | NA | 7 |
| Škavić 2015 | 2 | 1 | 1 | 1 | 1 | 0 | 0 | 1 | NA | NA | NA | NA | 7 |
| Durakovic 2012 | 2 | 1 | 1 | 1 | 1 | 0 | 0 | 1 | NA | NA | NA | NA | 7 |
| Harding 2006 | 2 | 1 | 1 | 1 | 1 | 0 | 0 | 1 | NA | NA | NA | NA | 7 |
| CDC 1996 | 2 | 1 | 1 | 1 | 1 | 0 | 0 | 1 | NA | NA | NA | NA | 7 |
| Goodson 1993 | 2 | 1 | 1 | 1 | 1 | 0 | 0 | 1 | NA | NA | NA | NA | 7 |
| **Others** | | | | | | | | | | | | | |
| Windsor 2020 | 2 | 1 | 1 | 1 | 1 | 0 | 0 | 1 | NA | NA | NA | NA | 7 |
| Brenner 2009 | 2 | 2 | 1 | 2 | 1 | 0 | 0 | 1 | 2 | 1 | 1 | 2 | 15 |
| Broi 2009 | 2 | 0 | 0 | 1 | 1 | 0 | 0 | 0 | NA | NA | NA | NA | 4 |

Notes: MINORS, methodological index for non-randomized studies; NA, non-applicable; 0(not reported); 1(inadequate reported); 2 (adequate reported).

**Supplemental Table 2** Characteristics of Swimmers Who Died of Cardiovascular Diseases

| **Cardiovascular diseases** | **Windsor 2020 (n=2)** | **Harris 2017 (n=27)** | **Kurosu 2016 (n=1)** | **Škavić 2015 (n=1)** | **Anästhesie 2015 (n=1)** | **Duraković 2012 (n=17)** | **Duraković 2011 (n=6)** | **Cedrone 2010 (n=1)** | **Tester 2009 (n=28)** | **Durakovic 2008 (n=1)** | **Duraković 2004 (n=1)** | **Ackerman 1999 (n=1)** | **Myrianthefs 1997 (n=1)** | **Janataa 1994 (n=1)** |
| --- | --- | --- | --- | --- | --- | --- | --- | --- | --- | --- | --- | --- | --- | --- |
| Sex |  |  |  |  |  |  |  |  |  |  |  |  |  |  |
| Male | 2 | NR | 1 | 0 | 1 | 16 | 6 | 1 | 20 | 1 | 1 | 0 | 0 | 0 |
| Female | 0 | NR | 0 | 1 | 0 | 1 | 0 | 0 | 8 | 0 | 0 | 1 | 1 | 1 |
| Age |  |  |  |  |  |  |  |  |  |  |  |  |  |  |
| Mean±SD | 43.5±2.1 | NR | 17.0±0 | 82.0±0 | 11.0±0 | 59.2±19.2 | 76.2±6.1 | 20.0±0 | 15.7±6.9 | 18.0±0 | 18.0±0 | 19.0±0 | 12.0±0 | 16.0±0 |
| Range | 42-45 | NR | 17 | 82 | 11 | 18-82 | 68-82 | 20 | 4-39 | 18 | 18 | 19 | 12 | 16 |
| Body of water |  |  |  |  |  |  |  |  |  |  |  |  |  |  |
| pool | NR | NR | NR | 0 | 0 | 2 | 1 | 0 | NR | NR | NR | 1 | NR | 0 |
| Lake/reservoir | 1 | NR | NR | 0 | 1 | 1 | 0 | 0 | NR | NR | NR | 0 | NR | 0 |
| Sea/harbor | NR | NR | NR | 1 | 0 | 12 | 5 | 0 | NR | NR | NR | 0 | NR | 0 |
| River | NR | NR | NR | 0 | 0 | 2 | 0 | 1 | NR | NR | NR | 0 | NR | 1 |

**Supplemental Table 3** Characteristics of Swimmers

Who Died of Cerebrovascular Diseases

| **Cerebrovascular diseases** | **Windsor**  **2020**  **(n=1)** | **Durakovic 2012**  **(n=1)** |
| --- | --- | --- |
| Sex |  |  |
| Male | 1 | 1 |
| Female | 0 | 0 |
| Age |  |  |
| Mean±SD | 48.0±0 | 61.0±0 |
| Range | 48 | 61 |
| Body of water |  |  |
| pool | NR | 0 |
| Lake/reservoir | NR | 0 |
| Ocean/harbor | NR | 1 |
| River | NR | 0 |

**Supplemental Table 4** Characteristics of Swimmers

Who Died of Respiratory Diseases

| **Respiratory diseases** | **Boyd**  **2015**  **(n=4)** | **Diamond 2011**  **(n=1)** |
| --- | --- | --- |
| Sex |  |  |
| Male | 3 | 1 |
| Female | 0 | 0 |
| Age |  |  |
| Mean±SD | NR | 18.0±0 |
| Range | 17-22 | 18 |
| Body of water |  |  |
| pool | NR | 0 |
| Lake/reservoir | NR | 1 |
| Ocean/harbor | NR | 0 |
| River | NR | 0 |

**Supplemental Table 5** Characteristics of Swimmers Who Died of Hazardous Conditions

| **Hazardous conditions** | **Knechtle 2020**  **(n=2)** | **Atilgan 2020 (n=1)** | **Focardi 2019 (n=1)** | **Sinton 2016 (n=4)** | **Škavić 2015 (n=1)** | **Durakovic 2012 (n=1)** | **Harding 2006 (n=1)** | **CDC 1996 (n=1)** | **Goodson 1993**  **(n=1)** |
| --- | --- | --- | --- | --- | --- | --- | --- | --- | --- |
| Sex |  |  |  |  |  |  |  |  |  |
| Male | 2 | 0 | 1 | NR | 0 | 1 | 0 | 1 | 1 |
| Female | 0 | 1 | 0 | NR | 1 | 0 | 1 | 0 | 0 |
| Age |  |  |  |  |  |  |  |  |  |
| Mean±SD | 53.0±2.0 | 12.0±0 | 13.0±0 | NR | 66.0±0 | 19.0±0 | 20.0±0 | 13.0±0 | 13.0±0 |
| Range | 51-55 | 12 | 13 | NR | 66 | 19 | 20 | 13 | 13 |
| Body of water |  |  |  |  |  |  |  |  |  |
| pool | NR | 1 | 1 | NR | 0 | 0 | 0 | 0 | 1 |
| Lake/reservoir | NR | 0 | 0 | NR | 0 | 0 | 1 | 1 | 0 |
| Ocean/harbor | NR | 0 | 0 | NR | 1 | 1 | 0 | 0 | 0 |
| River | NR | 0 | 0 | NR | 0 | 0 | 0 | 0 | 0 |

**Supplemental Table 6** Characteristics of Swimmers Who Died of Drowning

Due to Other or Unknown Causes

| **Others** | **Windsor**  **2020**  **(n=2)** | **Brenner**  **2009**  **(n=88)** | **Broi**  **2009**  **(n=1)** |
| --- | --- | --- | --- |
| Sex |  |  |  |
| Male | 1 | 59 | 1 |
| Female | 1 | 29 | 0 |
| Age |  |  |  |
| Mean±SD | 45.5±16.3 | 6.1±6.1 | 50.0±0 |
| Range | 34-57 | 1-19 | 50 |
| Body of water |  |  |  |
| pool | NR | 57 | 1 |
| Lake/reservoir | NR | 15 | 0 |
| Ocean/harbor | NR | NR | 0 |
| River | NR | NR | 0 |
